# Stimulation during Anaesthesia

**Published:** 1910-04

**Authors:** W. A. Selman

**Affiliations:** Atlanta, Ga.


### STIMULATION DURING ANAESTHESIA.

BY W. A. SELMAN, M. D., ATLANTA, GA.

The Anaesthetist should be able to administer any ordinary anaesthetic requested by the surgeon, and doing this, he should know the dangers of each, and how to recognize them in time to prevent any interference with the operation due to the anaesthesia.

To do this requires the constant attention of the anaesthetist to his patient from the time anaesthesia is begun until the patient is put to bed, and under the care of a nurse.

The majority of cases, especially in hospitals and sanatoria, where the patient has been prepared by laxative and dietetic treatment, will take the anaesthetic quietly and smoothly without any stimulation, yet in that minority of cases who do not take anaesthesia well, and emergency cases where no preparation has been possible, will be found some cases that will tax the ingenuity of the anaesthetist to the utmost, to know what is best to give the patient to produce an even anaesthesia, or maybe to prevent a sudden collapse.

Stimulation, so far as the anaesthetist is concerned, begins just before the time of operation, or half to one hour beforehand, and ends when he has turned the patient over to a nurse or someone responsible for his welfare. Whatever stimulation before this, whether in the form of diet or medicine; or whatever stimulation is to be kept up after the operation, is of course under the orders of the surgeon. But when the anaesthetist begins his work, he is usually the only physician present, the surgeon and assistants being busy "scrubbing up" in some other room, and in view of the fact that many of the serious results of anaesthesia have occurred in the early stages, it is imperative that the anaesthetist should know the signs of danger from whatever anaesthetic used, and learn to act on his own responsibility at once instead of waiting for help and advice when it may be too late to avail when he gets it.

Anticipation is as much the anaesthetist's duty as that of the assistant surgeon. For anticipating a danger and then avoiding it is better than to allow something to happen and then repair it. In this sense, stimulation is taken to mean, anything that betters the condition of the patient, whether mechanical or medicinal.

Often a timely change of position; a mere turning of the head to one side, a pushing forward of the jaw, or a lowering of the head will suffice to re-establish a uniform respiration when hypodermic medication would be a loss of time and useless. In fact, hypodermic medication has its greatest field of usefulness in anticipation of danger, and when administered in time, sustains the patient and prevents a collapse.

This prevention of accidents is what the anaesthetist is to look out for, as well as to revive them when serious trouble occurs. In discussing these dangers and how to prevent them, we will consider only the two anaesthetics most commonly used, Chloroform and Ether, whether given singly or in sequence.

These two substances are different chemically, act differently physiologically and exhibit their dangers in different ways. The records show that the immediate dangers from the chloroform are from five to six times those of ether, but these records are made up from cases reported from different parts of the country, and includes cases given by unskilled as well as by skilled anaesthetists. I believe that with skilled anaesthetists, the difference in mortality would not be so great. However, chloroform acts as a depressant on the heart muscle, and unless freely diluted with air or oxygen, its vapor is not slow in exerting its depressing influence. It is not so irritating to the air passages as ether, so the increase in mucous is not so profuse. The stage of excitement is not so pronounced, and more often is exhibited as random talking than in struggling.

If the patient has been prepared for the anaesthetic by a restricted diet, usually there will be no occasion for stimulation to lessen the secretion of mucous and to stimulate the heart against the depression. Atropin Sulph. gr. 1 150 to an adult, will be all the stimulation necessary. Should it be an emergency case with a full stomach, the stomach tube may be used to good advantage. Should the patient be already weak, such stimulation as would be indicated in any further strain on the system, should be given before anaesthesia. Especially is this a varied problem in emergency operations where the patients are already almost exsanguinated or in various degrees of intoxication with the elimentary canal distended with food. In such cases vomiting and uneven breathing are more frequent, and more danger from drawing some of the vomitus into the lower respiratory tract.

The administration of 1-6 gr. morphine often enables the patient to breathe quietly until the operation is over. Should the circulation at any time during chloroform administration show signs of weakening, discontinue it at once, and give an immediate hypodermic of strychnine 1-30 to 1-20 gr. with nitroglycerine gr. 1-50.

Should a collapse be threatened, besides allowing the patient fresh air, and keeping the respiratory passages as clear as possible, a continuous stream of oxygen gas should be administered either under an Esmark Inhaler by a tube passing through the gauze covering, or a nozzle fitting into one nostril. This I consider the quickest way of resusticating a patient and preventing a complete cessation of breathing. In conjunction with this, the patient may be hastily put in the Trendelenberg position without any loss of time, and with this mechanical procedure alone, the increased amount of blood to the upper extremity will accelerate the respiration, and with it the circulation. Besides furnishing a better drain of any mucous or vomitus obstructing the air passages. Should the patient be a child a quick lowering of the head by grasping the legs, and holding the patient's head downward will do the same.

Chloroform in full doses is a decided vasodilator, and effects not only the blood vessels proper, but the heart muscle itself. Should respiration cease altogether, artificial respiration should be begun immediately, and should be persisted in regularly and for a long time if respiration is not immediately restored.

In giving chloroform I prefer the Esmark Inhaler covered with half dozen layers of gauze, and a drop-bottle that will drop instead of pour. If the inhaler be never allowed to rest on the face, but constantly held a short distance away, thus at all times allowing a mixture of air, and entirely removing the mask when a deep inspiration is taken, as in coughing or during any sudden change in the respiration, as when the sphincter-ani is dilated. These simple precautions, remembering at the same time not to try to push a chloroform anaesthesia even though the patient be a screaming child, alarming a whole neighborhood, will save the anaesthetist a picture that never fades from his memory—a blanched face with a dull stare from fixed eyes.

In giving ether, the same amount of care should be taken, though its administration is not fraught with so much danger as that of chloroform. Here the preparation of the patient, by the administration of 1-150 gr. atropin half hour before hand, assists materially in checking the abundant flow of mucous, which is the most annoying and constant factor in the ether anaesthesia. Morphine Sulph. gr. 1-8-1-4 given at the same time assists in producing a very quiet, even anaesthesia with little mucous, and with the stage of excitement considerably lessened. However, I do not make it a routine procedure to use the morphine, for, should there be depression from any cause, it would be difficult to tell how much was attributed to that source. Neither do I make a routine of giving Hyoscine or Scopolamine for the same reason. They produce a narcosis that so resembles a depression that it is with difficulty you can see the respiratory movements, and to me that is an important guide.

Any one, to have the desired effect, should be given 30 minutes before anaesthesia is begun, for it is usually during the first stage of anaesthesia that the patient is most liable to strangulation from an excess of mucous, and a hypodermic given at that time is of no avail until several minutes later, even if the circulation be good, and a longer time ordinarily on account of the resultant cyanosis from occluded respiration.

When administering ether great care should be taken not to give it too freely at first, for suddenly giving strong fumes of ether is bound to meet with resistance from the patient, and excites an excessive flow of mucous and saliva in a few seconds, and when a patient is drenched with ether, at first they very often breathe badly throughout the anaesthesia. Besides, at this stage of anaesthesis the patient has not entirely lost consciousness, and keep the jaws so tightly clenched that it is with great difficulty that they can be opened, and then only with a screw gag, or wooden wedge, that the tongue may be drawn slightly forward or mucous sponged from the mouth. When this is the condition it is much better to withhold the anaesthetic until the patient is sufficiently conscious to expel the mucous from the throat than to push the ether in attempting to overcome the resistance by deep anaesthesia, for the cyanosis increases, and to it is added Stertor, which is always an indication of deep anaesthesia, and a timely warning to lessen the amount of ether, and give more air.

During the anaesthesia the head should be kept to one side, so that the mucous can drain into the hollow of the cheek and be sponged out.

The circulation varies with the respiration, and any obstruction or cessation of the respiration increases the pulse rate, and weakens its force. However, the heart continues to beat for some time after respiration ceases from ether. So when death seems impending recovery can generally be brought about by promptly leaving off the ether and giving artificial respiration alone or combined with the administration of oxygen.

In long continued operations where there is considerable loss of blood, or surgical shock, the pulse becomes rapid and shallow, and the respiration correspondingly so. To strengthen and support the circulation and with it the respiration, I have found strychnine gr. 1-30 1-20 the most reliable.

In weak and depleted patients the infusion of warm normal Saline solution acts as a general stimulant, and keeps up the pulse and Nitrous Oxide gas by using the Bennett Inhaler, but also use the chloroform-ether sequence, when it is preferred.

When anaesthesia is established by the gas-ether sequence, I change to the Esmark inhaler, and give by the open drop method.

In giving any anaesthetic the degree of anaesthesia will depend upon the operation and condition of the patient during same, from causes other than the anaesthesia. Much of the stimulation necessary is from these causes.

In cases of severe hemorrhage I have given 30 Minims of Adrenalin Chloride with fair results as a stimulant, but cannot say how much haemostatic result was obtained. I usually keep one hand on the facial artery, and at the same time press the jaw forward if necesary for good breathing.

In conclusion, will say take time in producing anaesthesia.

Second—Give plenty of air with chloroform or ether.

Third—Never push chloroform.

Fourth—Have a cylinder of Oxygen at hand ready for use, use it freely when indicated.

Fifth—Keep your head in all emergencies.

